# Serum immune markers and disease progression in an incident Parkinson's disease cohort (ICICLE‐PD)

**DOI:** 10.1002/mds.26563

**Published:** 2016-03-21

**Authors:** Caroline H. Williams‐Gray, Ruwani Wijeyekoon, Alison J. Yarnall, Rachael A. Lawson, David P. Breen, Jonathan R. Evans, Gemma A. Cummins, Gordon W. Duncan, Tien K. Khoo, David J. Burn, Roger A. Barker, on behalf of the ICICLE‐PD study group

**Affiliations:** ^1^ John van Geest Centre for Brain Repair, Department of Clinical NeurosciencesUniversity of CambridgeCambridgeUK; ^2^ Institute of NeuroscienceNewcastle UniversityNewcastleUK; ^3^ Medicine for the ElderlyWestern General HospitalEdinburghUK; ^4^ School of MedicineGriffith UniversityAustralia

**Keywords:** Parkinson's disease, immune markers, biomarkers, longitudinal studies

## Abstract

**Background:**

The immune system is a promising therapeutic target for disease modification in Parkinson's disease (PD), but appropriate immune‐related biomarkers must be identified to allow patient stratification for trials and tracking of therapeutic effects. The objective of this study was to investigate whether immune markers in peripheral blood are candidate prognostic biomarkers through determining their relationship with disease progression in PD.

**Methods:**

Serum samples were collected in incident PD cases and age‐matched controls. Subjects were clinically evaluated at baseline and 18 and 36 months. Ten cytokines and C‐reactive protein were measured, with data reduction using principal‐component analysis, and relationships between component scores and motor (MDS Unified Parkinson's Disease Rating Scale — part 3) and cognitive (Mini Mental State Examination [MMSE]) measures of disease severity/progression were investigated.

**Results:**

TNF‐α, IL1‐β, IL‐2, and IL‐10 were higher in PD (n = 230) than in controls (n = 93), *P* ≤ 0.001). Principal‐component analysis of log‐transformed data resulted in a 3‐component solution explaining 51% of the variance. Higher “proinflammatory” and lower “anti‐inflammatory” component scores were associated with more rapid motor progression over 36 months (*P* < 0.05), and higher “proinflammatory” component scores were associated with lower MMSE at all times (*P* < 0.05). Multiple linear regression analysis with adjustment for covariates confirmed “anti‐inflammatory” component score was the strongest predictor of slower motor progression (β = −0.22, *P* = 0.002), whereas proinflammatory cytokines were associated with lower baseline MMSE (β = −0.175, *P* = 0.007).

**Conclusions:**

Serum immune marker profile is predictive of disease progression in PD and hence a potential prognostic biomarker. However, interventional trials are needed to clarify whether peripheral immune changes causally contribute to the progression of PD. © 2016 The Authors. Movement Disorders published by Wiley Periodicals, Inc. on behalf of International Parkinson and Movement Disorder Society

Parkinson's disease (PD) is a common neurodegenerative disorder, affecting approximately 2%‐3% of people older than age 65. Although dopaminergic treatments have symptomatic benefit for some of the motor symptoms of the disease, their long‐term use is problematic, and there is an urgent need to develop therapies to slow the underlying disease process and prevent progression to debilitating nondopaminergic complications including postural instability and dementia. Inflammation is a promising new therapeutic target for disease modification.

Inflammatory change has been clearly demonstrated in the PD brain, both at postmortem^1^, ^2^, ^3^, ^4^ and using [11C]‐PK11195 positron emission tomography (PET) imaging in vivo.^5^, ^6^, ^7^ Immune alterations have also been reported to occur in the periphery, with altered cytokine levels^8^ and monocyte^9^ and lymphocyte subsets.^10^, ^11^ Although often considered secondary to the primary disease process, several lines of evidence suggest that these observations reflect a direct pathogenic role for the immune system in PD: (1) immune manipulation in animal models of PD can alter disease susceptibility and severity,^4^, ^12^ (2) genetic studies in humans show a significant association between major histocompatibility complex genes (HLA‐DR) and PD risk,^13^, ^14^ and (3) epidemiological studies show that individuals who regularly take nonsteroidal anti‐inflammatory drugs have a reduced risk of developing PD.^15^ Although the literature to date has mainly focused on disease risk, an arguably more relevant question is whether the immune response influences the rate of disease progression following diagnosis, from a predominantly motor syndrome with Lewy body pathology largely restricted to the brain stem to a condition with marked gait and axial disturbance and ultimately dementia with widespread cortical pathology.^16^ This question is of particular importance because the most realistic window for intervention with an immunomodulatory disease‐modifying therapy would be in the early symptomatic phase. In addition, appropriate immune‐related biomarkers need to be identified to facilitate future trials of immuno‐modulatory therapies to delay disease progression in PD.

Although central “markers” of immune activation are available via PET neuroimaging, measurement of immune markers in peripheral blood represents a more accessible and practical method of studying and monitoring the immune response in PD. There is increasing evidence that peripheral immune changes are highly relevant in neurodegenerative disease. For example, in Alzheimer's disease, systemic infections and higher levels of serum TNF‐α are associated with faster rates of cognitive decline^17^ through a proposed mechanism of immune‐brain interaction leading to activation of already “primed” microglia in the brain and accelerated neurodegeneration.^18^ Furthermore cultured peripheral blood mononuclear cells from PD patients produce higher levels of inflammatory cytokines in response to stimulation with lipopolysaccharides compared with controls, and these cytokine levels correlate with motor severity of disease.^8^ More recently, an association between higher plasma C‐reactive protein (CRP) levels and more rapid motor progression in PD has also been reported.^19^

This study investigated a panel of immune markers in serum samples from a large incident PD cohort and an age‐matched control group, to ascertain whether there was any association with disease status and measures of disease severity and progression over a subsequent 36 months of follow‐up. Thus, for the first time we explore the profile and relevance of serum immune markers in newly diagnosed PD cases with the aim of further informing our understanding of the relationship between peripheral immune responses and disease progression, and evaluating whether these easily accessible markers may constitute useful biomarkers in PD.

## Methods

### Subjects

Subjects were participants in the Incidence of Cognitive Impairment in Cohorts with Longitudinal Evaluation in Parkinson's Disease study (ICICLE‐PD). The cohort comprised 262 newly diagnosed PD patients recruited between 2009 and 2011 from the community and outpatient clinics in Newcastle and Cambridge, United Kingdom, as well as unrelated control subjects of similar age (n = 99) recruited from the community. Details of recruitment of 219 ICICLE‐PD cases have been previously published.^20^ An additional 43 cases identified using the same recruitment methods who did not complete the full ICICLE‐PD neuropsychological/imaging protocol within 6 months of referral required for our previous study, but had sufficient clinical data for the current study are included here. Idiopathic PD was diagnosed according to United Kingdom Parkinson's Disease Society Brain Bank criteria.^21^ Peripheral blood samples were collected at enrollment. Subjects were clinically and neuropsychologically assessed at baseline and at 18‐ and 36‐month follow‐up visits. The study was approved by the Newcastle and North Tyneside Research Ethics Committee. All patients provided written informed consent.

### Assessments

Baseline assessments included disease duration, family history, medication history, and comorbid conditions. Comorbidity was quantified in terms of number of organ systems affected using the Cumulative Illness Rating Scale (CIRS; range, 0‐13).^22^ At each time, subjects were assessed with a number of standardized instruments including the Movement Disorder Society‐revised Unified Parkinson's Disease Rating Scale (MDS‐UPDRS),^23^ Mini Mental State Examination (MMSE),^24^ and Geriatric Depression Scale‐15 (GDS‐15).^25^ Levodopa‐equivalent daily doses (LEDDs) were calculated.^26^

### Measurement of Immune Markers

Venous blood samples were allowed to clot for 15 minutes prior to centrifugation at 2000 rpm for 15 minutes. Serum was removed and stored in 200‐μL aliquots at ‐80°C until assays were performed. A panel of key inflammation‐related markers was measured using Meso Scale Discovery (Rockville) electrochemiluminescent immunoassays, including the V‐PLEX human proinflammatory panel 1 (IFN‐γ, IL‐1β, IL‐2, IL‐4, IL‐6, IL‐8, IL‐10, IL‐12p70, IL‐13, and TNF‐α), and V‐PLEX human CRP. Assays were run according to the manufacturer's instructions. Samples were processed in duplicate.

### Statistical Analyses

Case‐control comparisons of clinical/demographic variables were performed using Student *t* tests for continuous variables and chi‐square tests or Fisher's exact test for categorical variables. Mean immune marker levels were compared using Mann‐Whitney *U* tests. Preliminary bivariate correlations between immune marker levels and clinical measures were assessed using Pearson's correlation coefficients.

Principal‐component analysis (PCA) was used to reduce redundancy in the immune marker data set. Resulting PCA component scores were extracted for analysis of association with motor (UPDRS‐III) and cognitive (MMSE) measures of disease severity and progression over time. Component scores were dichotomized at the mean into high and low groups to allow between‐group comparisons of motor scores (UPDRS III) and cognitive scores (MMSE) at the 3 assessment times using repeated‐measures analysis of variance (RMANOVA). Relationships between clinical measures and immune component scores were further evaluated using multiple linear regression analysis to allow for the effects of covariates. Dependent variables were UPDRS‐III, MMSE, or rate of change in these variables over time. In addition to immune component scores (as continuous variables), other covariates considered for inclusion in the regression models included age at study enrollment, age leaving full‐time education, sex, smoking status, comorbidity (CIRS systems score), use of anti‐inflammatory drugs, UPDRS III, MMSE, GDS‐15, and LEDD dose (all measured at baseline). Selection of variables for inclusion in the models was based on bivariate analyses (Pearson's correlations for continuous variables and Student *t* tests for categorical variables), with variables showing association at *P* < 0.10 with the dependent clinical outcome and PCA component scores selected for entry. All statistical analyses were conducted using SPSS software version 21.0

## Results

### Subjects

Of the 262 incident PD cases and 99 controls included at baseline, 11 cases were excluded because of a change in diagnosis at follow‐up, and serum samples were unavailable for 21 cases and 6 controls; thus, 230 cases and 93 controls were included. Follow‐up clinical and neuropsychological data were available for 209 cases and 81 controls at 18 months and 174 cases and 69 controls at 36 months. Demographic and clinical characteristics of subjects at baseline are presented in Table 1. Case and control groups were well matched, differing only in lower depression scores in the control cohort, as would be anticipated.

**Table 1:** Demographic and clinical characteristics of subjects at study enrollment

Variable	PD (n = 230)	Controls (n = 93)	*P*
Age at study enrollment (years)	66.4 (9.5)	68.0 (8.0)	0.118
Disease duration at study enrollment (years)	0.6 (0.5)		
Sex (% male)	61.7	52.7	0.134
Age leaving full‐time education (years)	17.8 (3.7)	18.0 (3.4)	0.604
UPDRS‐III	27.9 (11.5)		
MMSE	28.8 (1.3)	29.0 (1.2)	0.078
GDS‐15	2.9 (2.6)	1.0 (1.6)	< 0.001
LEDD (mg)	194.5 (161.5)		
CIRS systems score, median, (range)	2 (0–7)	3 (0–7)	0.070
Anti‐inflammatory drug use (%)	32.6	33.3	0.900

Values shown are mean (SD) unless otherwise stated. Continuous variables compared using Student *t* tests; categorical variables compared using chi‐square tests or Fisher's exact test as appropriate. UPDRS‐III, MDS Unified Parkinson's disease Rating Scale part 3; MMSE, Mini Mental State Examination; GDS‐15, Geriatric Depression Scale–15 item; LEDD, levodopa‐equivalent daily dose; CIRS, Cumulative Illness Rating Scale (number of organ systems affected).

### Immune Marker Levels

**Figure 1:**
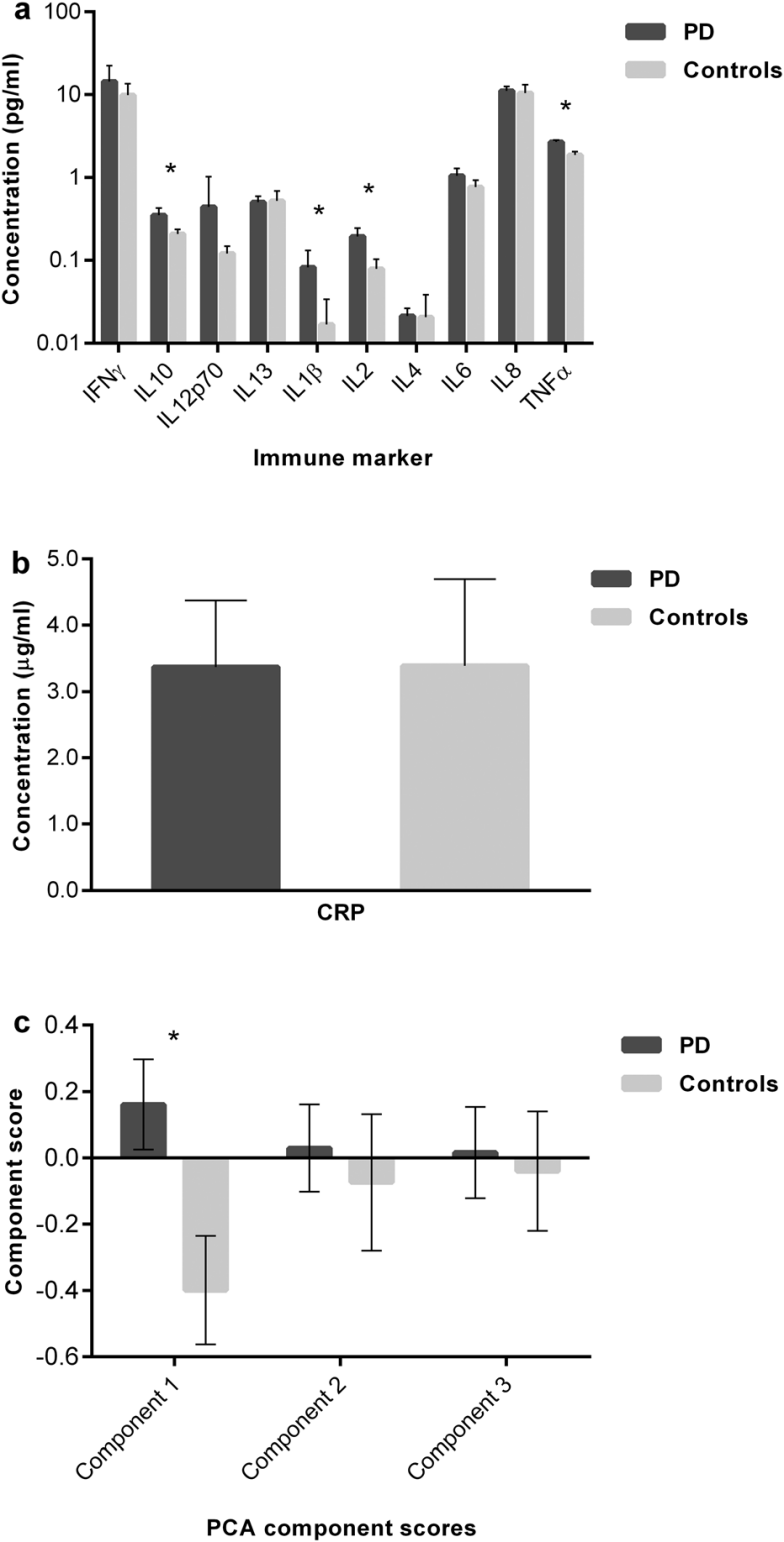
Comparison of immune marker profiles in cases (n = 230) and controls (n = 93). Bars represent mean values; error bars show 95% confidence intervals; *P < 0.001. (a) Cytokine levels (log scale); (b) CRP levels; (c) PCA‐derived component scores.

Comparison of cytokine concentrations in PD cases and controls revealed similar profiles in the 2 groups (Fig. 1a). Mean levels of TNF‐α, IL‐1β, IL‐2, and IL‐10 were higher in PD versus controls (*P* ≤ 0.001, Mann‐Whitney *U* test, withstanding Bonferroni correction for multiple testing; Supplementary Table 1). There were no differences in CRP levels between cases and controls. Prior to further analysis, immune marker and CRP data were log‐transformed using Ln(x + 1) to overcome right skewing of the data distributions without loss of zero data. Bivariate correlation analyses provided preliminary evidence of association between a number of immune markers and clinical measures (Supplementary Table 2). In the PD cohort, associations included IL‐6 with higher UPDRS‐III motor scores (*P* < 0.05), TNF‐α and CRP with faster rates of motor decline (change in UPDRS‐III per year), and IL‐13 with slower rate of motor decline (all *P* < 0.005); IFN‐γ, *P* < 0.005; TNF‐α, *P* < 0.005; IL‐6, *P* < 0.05; and CRP, *P* < 0.05 with lower MMSE scores and IL‐1β and IL‐2 with faster rate of cognitive decline (change in MMSE per year; both, *P* < 0.005). In the control group, there were bivariate correlations between IL‐1β, IL‐6, and IL‐8 and lower MMSE scores and between IL‐13 and slower rate of cognitive decline (albeit at a significance level of *P* < 0.05 and notwithstanding correction for multiple testing).

### PCA

The immune marker data set (log‐transformed) was examined for suitability for PCA. Inspection of the correlation matrix confirmed significant correlations (*r* > 0.2) for each variable other than IL‐8, and Bartlett's test of sphericity was statistically significant (*P* < 0.001), indicating that the data were likely factorizable. IL‐1β and IL‐8 were excluded, as Kaiser‐Meyer‐Olkin (KMO) measures were below 0.5, indicating sampling inadequacy. The overall KMO measure for the final analysis was 0.67.^27^ Three hundred thirteen subjects were included (following removal of outliers). PCA revealed 3 factors with Eigenvalues > 1, explaining 23.5%, 16.1%, and 11.3% of the variance. Inspection of the Scree plot also suggested 3 factors should be retained.^28^ The 3‐component solution explained 51% of the variance. Oblique oblimin rotation with Kaiser normalization was employed to aid interpretation. Component loading and communalities of the rotated solution are shown in Table 2. Interpretation of the data necessitates an oversimplification of the complex functions of these cytokines/markers but, broadly speaking, indicates 2 components that are mainly proinflammatory and one that is mainly anti‐inflammatory. Component 1 was designated “proinflammatory,” with the strongest loadings from IFN‐γ and TNF‐α, but also loadings from IL‐2 and IL‐10; component 2 was designated “anti‐inflammatory,” dependent on IL‐4 and IL‐13 as well as IL‐12p70; and component 3 was considered “proinflammatory”, depending principally on CRP and IL‐6.

**Table 2:** Rotated structure matrix for PCA of immune markers in 313 cases (post–outlier removal).

Inflammatory Marker	Rotated Component Coefficients			Communalities
	Component 1 Proinflammatory	Component 2 Anti‐inflammatory	Component 3 Proinflammatory	
IFN‐γ	*0.673*–	0.029	0.097	0.489
TNF‐α	*0.646*	−0.131	0.228	0.534
IL‐10	*0.591*	0.012	0.076	0.372
IL‐2	*0.570*	0.066	−0.144	0.326
IL‐4	−0.191	*0.702*	0.273	0.547
IL‐12p70	0.253	*0.670*	−0.096	0.540
IL‐13	0.020	*0.664*	−0.133	0.471
IL‐6	0.032	0.046	*0.808*	0.662
CRP	0.147	−0.049	*0.758*	0.643

Rotation method: Oblimin rotation with Kaiser normalization.

Major loadings for each component are in italic.

### Association Between Component Scores and Disease Measures

Comparison of mean immune component scores indicated that component 1 scores (proinflammatory) were significantly elevated in PD versus controls (*P *< 0.001; Student *t* test; Fig. 1c), but there were no significant between‐group differences for components 2 and 3 scores.

**Figure 2:**
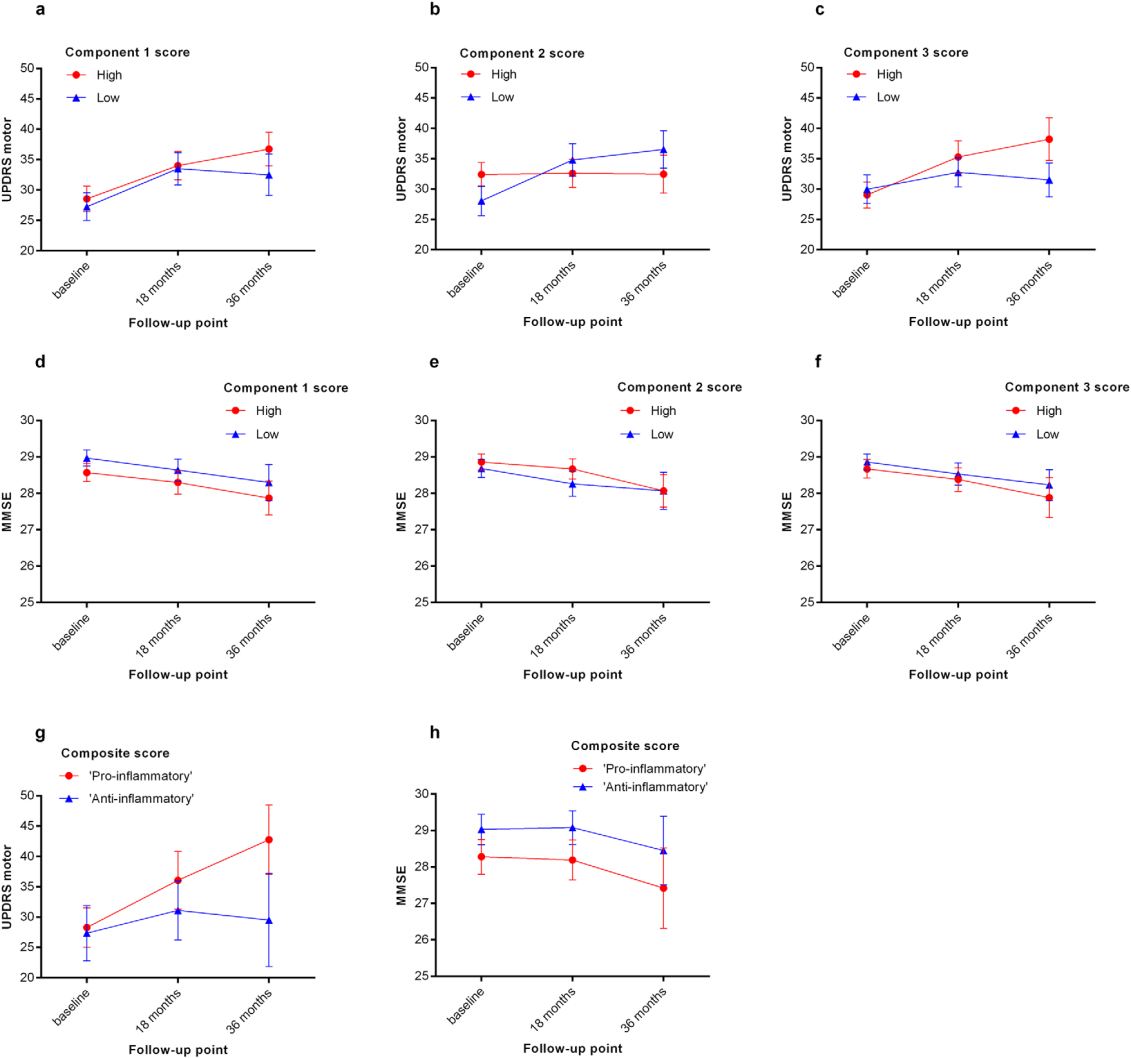
Longitudinal motor and cognitive parameters in the PD group (n = 230), stratified by immune component scores. Mean UPDRS‐III and MMSE scores and 95% confidence intervals are shown. Components 1 and 3 were designated as “proinflammatory” and component 2 was designated as “anti‐inflammatory.” (a‐c) UPDRS–III at baseline, 18 months, and 36 months in those with high (greater than group mean) versus low (less than group mean) component scores. (d‐f) MMSE scores at each time in those with high (more than group mean) versus low (less than group mean) component scores. (g) UPDRS‐III and (h) MMSE scores in those with a high overall proinflammatory index (components 1 and 3 scores > group mean, component 2 scores ≤ group mean, n = 32) versus a high overall anti‐inflammatory index (component score 2 > group mean, component scores 1 and 3 group ≤ mean, n = 26). [Color figure can be viewed in the online issue, which is available at wileyonlinelibrary.com.]

Component scores were dichotomized at the mean into high‐ and low‐score groups for comparison of longitudinal UPDRS‐III and MMSE scores within the PD group (Fig. 2). High component 1 scores (proinflammatory) were associated with worsening of UPDRS‐III scores over time (RMANOVA, component*time *F =* 3.80, *P* = 0.023). Component 2 scores (anti‐inflammatory) had an opposite effect, with UPDRS‐III remaining stable in those with high component 2 scores versus worsening over time in those with low scores (component*time *F* = 5.34, *P* = 0.005). Although UPDRS‐III scores at baseline appear to be paradoxically higher (worse motor function) in the group with high anti‐inflammatory component 2 scores (Fig. 2b), this difference was not statistically significant after correction for multiple testing and not confirmed in linear correlation analyses. High component 3 scores (pro‐inflammatory) were associated with higher UPDRS‐III scores overall (main effect, *F* = 8.46, *P* = 0.004). Patients were further stratified into those with a high overall proinflammatory index (components 1 and 3 scores above group mean, component 2 scores below group mean) versus a high overall anti‐inflammatory index (component score 2 above mean, component scores 1 and 3 below mean). There was a clear separation of these 2 groups over time in UPDRS‐III scores with progressive worsening in the high pro‐inflammatory index group (group*time *F* = 10.31, *P* < 0.001; Fig. 2g) and a clinically significant 13.3‐point between‐group difference at 36 months.

Analysis of MMSE scores in the PD group indicated an overall reduction over all 3 assessment times in those with high versus low component 1 scores (RMANOVA *F*= 6.03, *P* = 0.015), but there was no time‐dependent effect. No significant effects of component 2 or 3 scores on MMSE were demonstrable. Comparison of groups with a high overall proinflammatory index versus a high overall anti‐inflammatory index (as above) confirmed lower mean MMSE scores in the proinflammatory group across all times (*F* = 5.31, *P* = 0.026; Fig 2h) but with a relatively modest effect size (0.75‐1.0 points). There was no significant effect of component scores on MMSE scores in the control group.

Associations between disease measures and immune component scores were further evaluated using multiple linear regression analyses to allow for correction for potential confounding variables. Based on the relationships identified in the RMANOVAs, which indicated that immune marker scores had an impact on change in UPDRS‐III over time, but an impact on MMSE that was not time dependent, 2 regression models were constructed, with change in UPDRS per year and MMSE (baseline) as dependent variables. Predictor variables included the 3 immune component scores, and potential covariates were selected for inclusion based on bivariate analyses (*P* < 0.10), as described in the Methods section. For motor progression (change in UPDRS III/year), variables included were immune component scores, age at study enrollment, comorbidity (CIRS system score), and use of anti‐inflammatory drugs. The resulting model significantly predicted motor progression (*F*_6,196_ = 4.64, *P* < 0.001) with anti‐inflammatory component 2 and proinflammatory component 3 having opposing effects in the model (β = ‐0.220, *P* = 0.002, and β = 0.121, *P* = 0.085, respectively), but there was no significant effect of proinflammatory component 1, and there were no significant covariate effects (*P* > 0.10; Supplementary Table 3). For MMSE, variables entered included immune component scores, age at study enrollment, age leaving full‐time education, and comorbidity (CIRS system score). The resulting model significantly predicted MMSE (*F*_6,216_ = 5.61, *P* < 0.001) with component 1 (β = −0.175, *P* = 0.007), age (β = −0.171, *P* = 0.012), and age leaving education (β = 0.198, *P* = 0.003) being significant contributors to the model, but the contributions of components 2 and 3 were nonsignificant (*P* > 0.10; Supplementary Table 4). For both models, assumptions of linearity, independence of errors, homoscedasticity, unusual points, and normality of residuals were met.

## Discussion

This is the first study to investigate serum immune markers in a large cohort of newly diagnosed PD patients, and demonstrates that the immune marker profile in early disease is associated with cognitive impairment and is predictive of the future rate of motor progression. A more proinflammatory profile is associated with lower MMSE scores and faster motor decline, whereas a more anti‐inflammatory profile is associated with better cognitive ability and stable motor function. The effect size of these immune factors is clinically significant, with a 13.3‐point difference in UPDRS‐III points between those with an overall proinflammatory versus an overall anti‐inflammatory profile (Fig. 2g), more than double the reported minimal clinically important change in the UPDRS‐III of 5 points.^29^ Our data are in keeping with the hypothesis that peripheral immune changes might influence disease progression in PD, although only interventional trials using immunomodulatory therapies will truly be able to demonstrate a causal link. Nonetheless, this study highlights the potential of serum immune markers as possible biomarkers in PD, which may help facilitate such trials.

Existing data on serum or plasma cytokines in PD is limited to a few reports of elevated cytokines (including TNF‐α, IL‐6, IL‐1, and IL‐2) in small cohorts of PD patients (n < 80) compared with controls^30^, ^31^, ^32^, ^33^ and 2 small cross‐sectional studies reporting association between IL‐6 and motor function^34^ and TNF‐α and the soluble IL‐2 receptor and nonmotor severity,^35^ respectively. However, it is not clear whether any such associations simply reflect a secondary effect of a more advanced disease state on the immune system. Longitudinal studies are necessary to investigate whether inflammatory marker changes predate a more aggressive disease course. Only 1 previous study, to our knowledge, has adopted such a longitudinal approach, investigating the role of CRP in 375 PD cases and reporting an association with more rapid motor deterioration (UPDRS‐III),^19^ which is in keeping with our findings.

Although studies to date have generally adopted a highly selective approach in the immune markers measured, this study has taken a more unbiased data‐driven approach through measuring a panel of 11 markers with both pro‐ and anti‐inflammatory effects and using PCA to identify patterns associated with disease state and severity. The interpretation of the components identified through PCA is not straightforward, given the complexity of multiple functions attributed to different cytokines,^36^ but nonetheless clear patterns emerged from our data. Component 1 was mainly driven by IFN‐γ and TNF‐α, key proinflammatory cytokines that mediate a Th1‐type cell‐mediated immune response. IL‐2 and IL‐10 also contributed to component 1. IL‐2 has an accepted proinflammatory role in the cell‐mediated immune response. Although IL‐10 is generally considered to have predominantly anti‐inflammatory effects, its bioactivity is highly complex, with roles in costimulation of B cells, NK cells, and certain types of CD‐8 T cells and production of IFN‐γ.^37^ Component 2 had significant loadings from IL‐4 and IL‐13, which have similar anti‐inflammatory effects on macrophages, suppressing IFN‐γ production, and play an important role in the Th2‐type humoral immune response. However, IL‐12p70 also loaded onto this component despite its generally accepted proinflammatory role.^36^ Component 3 was dependent on IL‐6 and CRP, 2 key proinflammatory markers that are closely interrelated, with IL‐6, being an important inducer of hepatic synthesis of CRP. Their segregation from proinflammatory/Th1 component 1 was in keeping with the known role of IL‐6 in skewing the immune response from Th1 toward Th2 via inhibition of IFN‐γ production.^38^

Our data suggest that these 3 components of the peripheral inflammatory/immune response can be segregated not only in terms of their constituent markers, but also in terms of their association with PD. Component 1 was associated with the disease state itself, being higher in patients than in controls and associated with lower baseline MMSE scores in the PD group, suggesting that it may simply be a secondary response to the degree of neurodegenerative pathology. Conversely, component scores 2 and 3 were not associated with disease status or MMSE but had prognostic value in terms of motor progression independent of any covariate effects; the effects were in opposing directions, as might be anticipated, with component 2 associated with slower motor progression and component 3 with more rapid motor progression. Thus, some of the immune marker variation in different individuals with PD might reflect intrinsic differences in the proinflammatory/anti‐inflammatory balance of the peripheral immune response, independent of baseline disease state. However, it is also possible that lack of association with baseline clinical state is because of a time lag between clinical measures of the disease and underlying pathological change. The underlying stimulant(s) of the peripheral immune response in PD remain unclear, but abnormal α‐synuclein aggregates in the periphery may be involved.^39^ Systemic infections might also be relevant (particularly in the case of component 3: IL‐6/CRP). Although comorbidity was not a significant confounding factor in our analyses, comorbidity scores do not reflect minor infections around the time of sampling, which this study did not capture.

Strengths of this study include the large unselected cohort of PD cases recruited close to diagnosis, a well‐matched control cohort, measurement of a comprehensive panel of pro‐ and anti‐inflammatory markers with a data‐driven approach to identify relevant variation, and the availability of longitudinal follow‐up data to 36 months to allow identification of prognostic immune factors. Limitations include loss of variation in the immune marker data set, which is inherent to the PCA process, and attrition in the cohort over time because of death/loss to follow‐up/withdrawal, which is inevitable in longitudinal studies. Attrition rates were 24% in the PD group and 26% in the control group over 36 months of follow‐up, although between‐group comparisons of those assessed and not assessed at 36 months did not reveal any significant differences in terms of age, sex, disease duration, age leaving education, baseline UPDRS‐III, MMSE, GDS, LEDD, comorbidity score, anti‐inflammatory drug use, or immune component score (Supplementary Table 5), suggesting that any attrition bias is likely to have been minimal. A further limitation is that MMSE may not be sufficiently sensitive to change over a 36‐month period to allow us to capture any effect of the inflammatory profile on cognitive decline over time. Furthermore, the MMSE has shortcomings in measuring executive function, although it is recommended as a global screening tool for diagnosing dementia in PD.^40^ Further follow‐up of the cohort is ongoing and will ultimately allow us to evaluate whether immune markers have utility in predicting long‐term disease outcomes including dementia. It would also be of interest in future studies to explore whether the relevance of immune markers varies in different disease subtypes, for example, in monogenic forms of the disease associated with mutations in genes relevant to immune function such as *LRRK2*,^41^ or in individuals carrying PD‐associated polymorphisms in HLA‐DR.^13^

In summary, we present the first comprehensive evaluation of serum immune markers in newly diagnosed PD compared with controls and demonstrate contrasting associations between proinflammatory and anti‐inflammatory markers and subsequent disease course. Although not useful as diagnostic biomarkers, given the overlap between cases and controls, serum immune marker profiles warrant further investigation as potential prognostic biomarkers for patient stratification. In particular, once further longitudinal outcome data are available in this or similar cohorts, the next step would be to adopt a data modeling approach using pro‐ and anti‐inflammatory composite scores derived directly from measured immune marker levels to create a predictive model that can be converted into an algorithm for use in individual patients. In addition to its potential value in prognostication, this work is also of relevance to the debate on whether the peripheral immune balance between pro‐ and anti‐inflammatory responses has an etiological impact on disease progression in PD. Our data are supportive of such a hypothesis but cannot demonstrate a causal link, and interventional studies of immune‐modulating therapies are needed to address this question.

## Author roles

C.H. Williams‐Gray: study design, acquisition of data, analysis and interpretation of data, drafting of the manuscript. R. Wijeyekoon: acquisition and analysis of data, critical revision of the manuscript. A.J. Yarnall: acquisition of data, critical revision of the manuscript. R.A. Lawson: acquisition of data, critical revision of the manuscript. D.P. Breen: acquisition of data, critical revision of the manuscript. J.R. Evans: acquisition of data, critical revision of the manuscript. GA Cummins: acquisition of data, critical revision of the manuscript. G.W. Duncan: acquisition of data, critical revision of the manuscript. T.K. Khoo: acquisition of data, critical revision of the manuscript. D.J. Burn: study design, critical revision of the manuscript. R.A. Barker: supervision of study, study design, critical revision of the manuscript.

## Supporting information

Additional Supporting Information may be found in the online version of this article at the publisher's web‐site.

Supporting Information
