# Supplementary material for: Serum immune markers and disease progression in an incident Parkinson's disease cohort (ICICLE‐PD)
Source: Mov Disord. 2016 Mar 21;31(7):995–1003. doi: 10.1002/mds.26563 (PMC4957620; doi:10.1002/mds.26563)
Supplement: Supplementary file 1 — Supporting Information [file MDS-31-995-s001.docx]

**Supplementary tables**

**Supplementary table 1:** mean (SD) immune marker levels in cases and controls

| **Immune marker** | **Concentration (pg/ml)** | | ***p*** |
| --- | --- | --- | --- |
|  | **PD (n=230)** | **Controls (n=93)** |  |
| IFNγ | 14.43 (61.21) | 9.79 (18.02) | 0.96 |
| IL10 | 0.35 (0.61) | 0.21 (0.14) | 0.001 |
| IL12p70 | 0.44 (4.50) | 0.12 (0.13) | 0.40 |
| IL13 | 0.50 (0.71) | 0.52 (0.81) | 0.99 |
| IL1β | 0.08 (0.38) | 0.02 (0.08) | <0.001 |
| IL2 | 0.19 (0.39) | 0.08 (0.12) | <0.001 |
| IL4 | 0.02 (0.04) | 0.02 (0.08) | 0.19 |
| IL6 | 1.05 (1.85) | 0.77 (0.77) | 0.07 |
| IL8 | 11.11 (11.14) | 10.43 (12.12) | 0.72 |
| TNFα | 2.70 (1.19) | 1.89 (0.87) | <0.001 |
| CRP | 3368.98 (7761.38) | 3383.92 (6373.18) | 0.12 |

Between group comparisons using Mann Whitney U tests.

**Supplementary table 2:** correlation matrix showing bivariate correlations between immune marker levels and clinical measures

| **PD** | | | | | |
| --- | --- | --- | --- | --- | --- |
| **Immune marker** | **Age** | **UPDRS-III** | **Change in UPDRS-III per year** | **MMSE** | **Change in MMSE per year** |
| IFNγ | 0.02 | 0.05 | 0.11 | -0.20** | -0.13 |
| IL10 | 0.05 | 0.06 | -0.08 | -0.02 | 0.04 |
| IL12p70 | 0.00 | 0.05 | -0.11 | -0.11 | -0.10 |
| IL13 | -0.05 | 0.02 | -0.24** | 0.08 | 0.05 |
| IL1β | 0.13 | 0.10 | -0.08 | 0.01 | -0.26** |
| IL2 | 0.06 | 0.00 | 0.10 | -0.12 | -0.22** |
| IL4 | -0.02 | -0.01 | -0.14 | 0.12 | -0.12 |
| IL6 | 0.20** | 0.15* | 0.14 | -0.15* | -0.02 |
| IL8 | 0.08 | -0.04 | 0.05 | -0.04 | 0.60 |
| TNFα | 0.08 | 0.02 | 0.29** | -0.26** | 0.00 |
| CRP | 0.10 | 0.01 | 0.21** | -0.16* | -0.02 |
| **Controls** | | | | | |
| IFNγ | 0.13 |  |  | -0.13 | 0.10 |
| IL10 | 0.19 |  |  | -0.09 | 0.07 |
| IL12p70 | 0.08 |  |  | -0.10 | 0.08 |
| IL13 | -0.79 |  |  | -0.03 | 0.22* |
| IL1β | 0.03 |  |  | 0.11 | -0.04 |
| IL2 | 0.17 |  |  | -0.24* | 0.12 |
| IL4 | 0.04 |  |  | 0.09 | 0.02 |
| IL6 | 0.25* |  |  | -0.25* | 0.07 |
| IL8 | 0.26* |  |  | -0.28* | -0.07 |
| TNFα | 0.34** |  |  | -0.19 | -0.11 |
| CRP | 0.08 |  |  | -0.05 | -0.04 |

Pearson’s correlation coefficients; * denotes significance at p < 0.05 level, ** denotes significance at p < 0.005 level. Immune marker levels log transformed using using Ln(x+1) prior to analysis to overcome right skewing without loss of zero data.

**Supplementary table 3:** summary of multiple linear regression analysis with change in UPDRS-III per year as the dependent variable.

| **Variable** | **Unstandardised coefficient**  **B** | **SE(B)** | **Standardised coefficient**  **β** | **p** |
| --- | --- | --- | --- | --- |
| Constant | -1.958 | 2.474 |  | 0.430 |
| Component 1 (‘pro-inflammatory’) | 0.104 | 0.320 | 0.022 | 0.746 |
| Component 2 (‘anti-inflammatory’) | -1.063 | 0.333 | -0.220 | 0.002 |
| Component 3 (‘pro-inflammatory’) | 0.551 | 0.318 | 0.121 | 0.085 |
| Age | 0.061 | 0.038 | 0.114 | 0.115 |
| CIRS system score (co-morbidity) | 0.093 | 0.225 | 0.030 | 0.679 |
| Anti-inflammatory drug use | 0.797 | 0.742 | 0.077 | 0.284 |

In addition to immune component scores, covariates reaching a significance level of p<0.10 in bivariate analyses were chosen for inclusion as predictor variables in the model. Covariates were baseline measures. CIRS - Cumulative Illness Rating Scale (number of organ systems affected).

**Supplementary table 4:** summary of multiple linear regression analysis with baseline MMSE as the dependent variable.

| **Variable** | **Unstandardised coefficient**  **B** | **SE(B)** | **Standardised coefficient**  **β** | **p** |
| --- | --- | --- | --- | --- |
| Constant | 29.155 | 0.767 |  | <0.001 |
| Component 1 (‘pro-inflammatory’) | -0.214 | 0.079` | -0.175 | 0.007 |
| Component 2 (‘anti-inflammatory’) | 0.054 | 0.081 | 0.043 | 0.511 |
| Component 3 (‘pro-inflammatory’) | 0.010 | 0.082 | 0.008 | 0.902 |
| Age | -0.023 | 0.009 | -0.171 | 0.012 |
| CIRS system score (comorbidity) | -0.028 | 0.055 | -0.034 | 0.613 |
| Age leaving full time education | 0.068 | 0.023 | 0.198 | 0.003 |

In addition to immune component scores, covariates reaching a significance level of p<0.10 in bivariate analyses were chosen for inclusion as predictor variables in the model. Covariates were baseline measures. CIRS - Cumulative Illness Rating Scale (number of organ systems affected).

**Supplementary table 5:** Comparison of baseline demographic and clinical characteristics, and immune component scores in subjects followed up at 36 months versus subjects not reassessed.

|  | **PD** | | | **Controls** | | |
| --- | --- | --- | --- | --- | --- | --- |
|  | **Assessed** | **Not assessed** | **p** | **Assessed** | **Not assessed** | **p** |
| **n** | 174 | 56 |  | 69 | 24 |  |
| **Age** | 66.04 (9.14) | 67.54 (10.47) | 0.30 | 68.08 (7.10) | 67.94 (10.41) | 0.94 |
| **Gender**  **(% male)** | 53.57 | 64.3 | 0.15 | 50.72 | 58.33 | 0.52 |
| **Disease duration** | 0.63 (0.49) | 0.64 (0.73) | 0.91 |  |  |  |
| **Age leaving education** | 17.93 (3.67) | 17.43 (3.84) | 0.38 | 18.32 (3.71) | 17.25 (2.45) | 0.19 |
| **UPDRS-III** | 27.24 (10.96) | 29.98 (13.21) | 0.12 |  |  |  |
| **MMSE** | 28.79 (1.18) | 28.63 (1.54) | 0.41 | 29.13 (1.19) | 28.71 (1.23) | 0.14 |
| **GDS-15** | 2.80 (2.61) | 3.00 (2.75) | 0.62 | 0.97 (1.48) | 1.04 (1.83) | 0.85 |
| **LEDD** | 195.84 (163.61) | 190.55 (155.94) | 0.83 |  |  |  |
| **CIRS**  **co-morbidity score**  **(median, range)** | 2 (0-7) | 2 (0-7) | 0.33 | 3 | 2 | 0.87 |
| **Anti-inflammatory drug use (%)** | 35.63 | 23.21 | 0.09 | 34.78 | 29.16 | 0.62 |
| **Component 1 score** | 0.18 (1.08) | 0.05 (0.85) | 0.36 | -0.43 (0.70) | -0.32 (0.99) | 0.58 |
| **Component 2 score** | 0.03 (0.99) | 0.04 (1.07) | 0.92 | -0.12 (0.91) | 0.06 (1.17) | 0.43 |
| **Component 3 score** | 0.03 (1.08) | -0.02(0.96) | 0.78 | -0.08 (0.85) | 0.79 (0.90) | 0.44 |

Mean (SD) values are shown unless otherwise indicated. UPDRS-III – MDS Unified Parkinson’s disease Rating Scale part 3; MMSE – Mini Mental State Examination; GDS-15 – Geriatric Depression Scale-15 item; LEDD – Levodopa equivalent daily dose; CIRS - Cumulative Illness Rating Scale (number of organ systems affected).
